# Modular genome-wide gene expression architecture shared by early traits of osteoporosis and atherosclerosis in the Young Finns Study

**DOI:** 10.1038/s41598-021-86536-0

**Published:** 2021-03-29

**Authors:** Binisha H. Mishra, Pashupati P. Mishra, Emma Raitoharju, Saara Marttila, Nina Mononen, Harri Sievänen, Jorma Viikari, Markus Juonala, Marika Laaksonen, Nina Hutri-Kähönen, Mika Kähönen, Olli T. Raitakari, Terho Lehtimäki

**Affiliations:** 1 Department of Clinical Chemistry, Faculty of Medicine and Health Technology, Tampere University, Tampere, Finland; 2 Finnish Cardiovascular Research Center Tampere, Faculty of Medicine and Health Technology, Tampere University, Tampere, Finland; 3 Department of Clinical Chemistry, Fimlab Laboratories, Tampere, Finland; 4 Gerontology Research Center (GEREC), Tampere University, Tampere, Finland; 5 The UKK Institute for Health Promotion Research, Tampere, Finland; 6 Department of Medicine, University of Turku, Turku, Finland; 7 Division of Medicine, Turku University Hospital, Turku, Finland; 8 Research Centre of Applied and Preventive Cardiovascular Medicine, University of Turku, Turku, Finland; 9 Fazer Lab Research, Oy Karl Fazer Ab, Helsinki, Finland; 10 Department of Paediatrics, Tampere University Hospital, Faculty of Medicine and Health Technology, Tampere University, Tampere, Finland; 11 Department of Clinical Physiology, Tampere University Hospital, Tampere, Finland; 12 Department of Clinical Physiology and Nuclear Medicine, Turku University Hospital, Turku, Finland; 13 Centre for Population Health Research, University of Turku and Turku University Hospital, Turku, Finland

**Keywords:** Computational biology and bioinformatics, Molecular biology, Systems biology

## Abstract

We analysed whole blood genome-wide expression data to identify gene co-expression modules shared by early traits of osteoporosis and atherosclerosis. Gene expression was profiled for the Young Finns Study participants. Bone mineral density and content were measured as early traits of osteoporosis. Carotid and bulbus intima media thickness were measured as early traits of atherosclerosis. Joint association of the modules, identified with weighted co-expression analysis, with early traits of the diseases was tested with multivariate analysis. Among the six modules significantly correlated with early traits of both the diseases, two had significant (adjusted p-values (p.adj) < 0.05) and another two had suggestively significant (p.adj < 0.25) joint association with the two diseases after adjusting for age, sex, body mass index, smoking habit, alcohol consumption, and physical activity. The three most significant member genes from the significant modules were NOSIP, GXYLT2, and TRIM63 (p.adj ≤ 0.18). Genes in the modules were enriched with biological processes that have separately been found to be involved in either bone metabolism or atherosclerosis. The gene modules and their most significant member genes identified in this study support the osteoporosis-atherosclerosis comorbidity hypothesis and can provide new joint biomarkers for both diseases and their dual prevention.

## Introduction

Cardiovascular disease and osteoporosis each contribute to a significant disease burden worldwide. It is estimated that roughly 17.9 million people died from cardiovascular disease in 2016, which accounts for 31% of all deaths globally^1^. The estimated number of fragility fractures in the EU in 2010 was 3.5 million, and it is predicted to rise by 28%, reaching 4.5 million, by 2025^2^. Osteoporosis and atherosclerosis share a similar pathophysiological mechanism that involves inflammatory cytokines and oxidized lipids^3^. The two diseases also share several risk factors, such as oestrogen deficiency, vitamin D abnormalities, dyslipidaemia, dietary calcium, dietary saturated fat and oxidative stress, as well as genetic biomarkers, such as osteoprotegerin, apolipoprotein E and the matrix gla protein^3,4^. Plasma lipids such as cholesterol, which has been shown to be associated with atherosclerosis, have also been shown to be associated with bone health^5,6^. In one of our previous studies, we identified three lipidome-based molecular triglycerides jointly associated with early traits of both osteoporosis and atherosclerosis^7^. Statin, a cholesterol-lowering drug for individuals who are at a high risk of cardiovascular disease, has also been shown to improve bone density^8^. Despite the strong evidence supporting the comorbidity hypothesis, studies investigating the underlying molecular mechanisms of the diseases using omics data, such as transcriptomics, are scarce.

Several transcriptomic studies have been previously carried out on osteoporosis^9,10^ and atherosclerosis^11,12^ independently. However, multivariate transcriptomics studies investigating the joint association of genes with both osteoporosis and atherosclerosis are lacking. While such multivariate transcriptomics studies are important, the analysis of a large number of genes in such high-dimensional data requires a multitude of tests and, consequently, multiple-testing correction, leading to the exclusion of genes with suggestive significance. However, a system-level bioinformatics approach largely reduces the multiple testing and increases the statistical power. We have recently used such an approach to identify lipidome-based molecular lipids jointly associated with early traits of osteoporosis and atherosclerosis^7^.

The objective of the present study was to perform a system-level analysis of genome-wide expression data in whole blood, used as a proxy for difficult-to-acquire samples such as bone or artery wall tissue, to identify gene co-expression modules, enriched pathways in the identified modules and the most important member genes jointly and significantly associated with the early traits of both osteoporosis and atherosclerosis. To achieve the stated goals, we used weighted gene co-expression network analysis (WGCNA)^13^, multivariate analysis of variance (MANOVA) and pathway analysis to identify gene modules and enriched pathways jointly associated with early traits of both osteoporosis and atherosclerosis.

## Material and methods

### Study participants

This study was based on the Young Finns Study (YFS), a prospective multi-centre follow-up study assessing cardiovascular risk factors from childhood to adulthood^14^. The study was initiated in 1980 with 3,596 children and adolescents aged 3–18 years. The participants were randomly selected from the areas of five university hospitals in Finland (Turku, Tampere, Helsinki, Kuopio, and Oulu) and have been followed up for over 40 years. The study was approved by the ethical committee of the Hospital District of Southwest Finland on 20 June 2017 (ETMK:68/1801/2017). All participants gave their written informed consent, and the studies were conducted in accordance with the Declaration of Helsinki. Data protection will be handled according to current regulations. The present study is based on 1,032 participants, aged 30–45 years, from the 2007 follow-up (Table 1), with four atherosclerotic and six osteoporotic traits as summarized in Table 2. The gene expression levels of the study participants were profiled based on the 2011 follow-up.

**Table 1:** Population characteristics of the Young Finns Study cohort. Data are expressed as means (± SD) or proportions (%).

	Men	Women
Number of subjects	454 (44%)	578 (56%)
Age, years	38 (± 5)	38 (± 5)
Body mass index, kg/m^2^	26.5(± 4)	25 (± 4.7)
Total cholesterol (mmol/l)	5.2 (± 0.9)	4.9 (± 0.8)
LDL cholesterol (mmol/l)	3.3 (± 0.8)	3.0 (± 0.7)
HDL cholesterol (mmol/l)	1.2 ± (0.3)	1.5 ± (0.3)
Triglycerides (mmol/l)	1.5 ± (0.7)	1.1 (± 0.5)
Serum glucose (mmol/l)	5.4 (± 0.5)	5.2 (± 0.7)
Insulin (IU/l)	10.4 (± 31.2)	8.0 (± 7.6)
C-reactive protein (mg/l)	1.7 (± 5.5)	1.9 (± 3.3)
Systolic blood pressure (mmHg)	124.8 (± 13.3)	116 (± 13.5)
Diastolic blood pressure (mmHg)	78.1 (± 11.1)	72.6 (± 10.9)
Alcohol consumption, units/day	1.4 (± 2.1)	0.6(± 0.7)
Physical activity index (MET-h/wk)	20.5 (± 22.70)	19.1 (± 20)
Daily smoking, %	77/453 (17%)	70/576 (12%)
Daily calcium intake (mg)	1393 (± 613)	1174 (± 453)
Daily vitamin D intake (μg)	8.6 (± 4.6)	7.4 (± 3.4)
Family risk factor for coronary heart disease (%)	72/454 (15.9%)	97/578 (16.8%)
Participants with osteoporosis (%)	3/451 (0.7%)	6/577 (1%)
Participants with epilepsy (%)	3/441 (0.7%)	4/573 (0.7%)
Participants with Crohn’s disease (%)	3/442 (0.7%)	5/573 (0.9%)
Participants with anorexia (%)	0	5/573 (0.9%)
Usage of corticosteroids at least once a month (%)	7/442 (2%)	37/573 (6%)
Participants with type 1 diabetes (%)	1/450 (0.2%)	4/578 (0.7%)
Participants with type 2 diabetes (%)	3/449 (0.7%)	3/578 (0.5%)
Participants with menopause (%)	–	0/578 (0%)

**Table 2:** Early traits of osteoporosis and atherosclerosis with their descriptive statistics among the study participants, expressed as mean ± SD.

Description (unit)	Acronym	Mean ± SD
**Early traits of atherosclerosis**		
Carotid intima-media thickness (average, mm)	*CIMTavg*	0.63 ± 0.10
Carotid intima-media thickness (maximum, mm)	*CIMTmax*	0.66 ± 0.11
Bulbus intima-media thickness (average, mm)	*BIMTavg*	0.80 ± 0.14
Bulbus intima-media thickness (maximum, mm)	*BIMTmax*	0.83 ± 0.14
**Early traits of osteoporosis**		
Distal radius trabecular bone mineral density (mg/cm^3^)	*DRTrD*	225 ± 36
Distal tibia trabecular bone mineral density (mg/cm^3^)	*DTTrD*	241 ± 34
Distal radius total bone mineral content (mg)	*DRToBMC*	245 ± 65
Radial shaft cortical bone mineral content (mg)	*RSCoBMC*	215 ± 45
Distal tibia total bone mineral content (mg)	*DTToBMC*	605 ± 127
Tibia shaft cortical bone mineral content (mg)	*TSCoBMC*	651 ± 110

### Assessment of early traits of atherosclerosis

Carotid and bulbus intima-media thickness (IMT) measurements were used as the early traits of atherosclerosis^7^. An ultrasound imaging device with a high-resolution system (Sequoia 512, Acuson) and 13.0 MHz linear array transducers was used for IMT measurements by trained sonographers following a standardized protocol. The image was focused on the posterior (far) wall, and images were recorded from the angle showing the greatest distance between the lumen–intima interface and the media–adventitia interface. A scan including the beginning of the carotid bifurcation and the common carotid artery was recorded and stored in digital format on optical discs for subsequent off-line analysis. All scans were analysed by one reader blinded to the participants’ details. The best-quality end-diastolic frame was selected. Several measurements of the common carotid far wall were taken approximately 10 mm proximally to derive the maximal carotid IMT. To assess the reproducibility of IMT measurements, we re-examined 60 participants 3 months after the initial visit (2.5% random sample). The between-visit coefficient of variation of IMT measurements was 6.4%. To assess the reproducibility of the IMT image analysis, 113 scans were re-analysed by a second observer, and the coefficient of variation was 5.2%. The mean and maximum carotid and bulbus IMTs were used in this study.

### Assessment of early traits of osteoporosis

The assessment of early traits of osteoporosis was based on peripheral quantitative computed tomography (pQCT) bone measurements from both the distal and diaphysis sites of the radius and tibia, as described elsewhere^7^. The tomographic slices were taken from the shaft (a cortical-rich bone site) and distal part (a trabecular-rich bone site) of the weight-bearing tibia (30% and 5% from the distal endplate of the tibia, respectively), and of the non-weight-bearing radius (30% and 4% from the distal endplate of the radius, respectively) according to our standard procedures^15^. For the shaft regions, the analysed bone traits were total area (ToA, mm^2^), cortical area (CoA, mm^2^) and cortical density (CoD, mg/cm^3^). For the distal parts of the radius and tibia, the measured bone traits were ToA (mm^2^), CoA (mm^2^) and trabecular density (TrD, mg/cm^3^). The in vivo precision of the used pQCT-measured traits ranged from 0.5% (CoD of the radial shaft) to 4.4% (CoA of the distal radius). Mineral content was calculated as 0.2 x (area/100) x density. The measured bone traits are shown in Table 2.

### Health and lifestyle data

The physical activity index, calculated as metabolic equivalents (METs) by combining information on the frequency, intensity and duration of physical activity, including leisure-time physical activity and commuting to the workplace (MET h/wk), was used to represent the physical activity of the participants. One MET corresponds to the energy consumption of one kilocalorie per kilogram of weight per one hour at rest^16^. Alcohol consumption was assessed from the participants’ reports on their alcohol consumption expressed in units (i.e., 14 g of alcohol) during the previous week^17^.

### Blood transcriptomic analysis

RNA isolation was performed from whole-blood samples collected from study participants during the 2011 follow-up. Expression levels were analysed with Illumina HumanHT-12 version 4 Expression BeadChip (Illumina Inc.), containing 47,231 expression and 770 control probes. Samples with fewer than 6,000 significantly detected expression probes (detection p-value < 0.01) were discarded. Raw Illumina summary probe-level data was exported from Beadstudio and processed in R (http://www.r-project.org/) using a nonparametric background correction, followed by quantile normalization with control and expression probes, with the *neqc* function in the limma package^18^ and a log2 transformation. Nine samples were excluded due to sex mismatch between the recorded sex and predicted sex based on *RPS4Y1-2* and *XIST* mRNA levels on the Y and X chromosomes, respectively. After quality control, expression data was available for 1,654 samples, including 4 technical replicates, which were used to examine batch effects and subsequently excluded before further analysis.

### Biostatistical analysis

**Figure 1:**
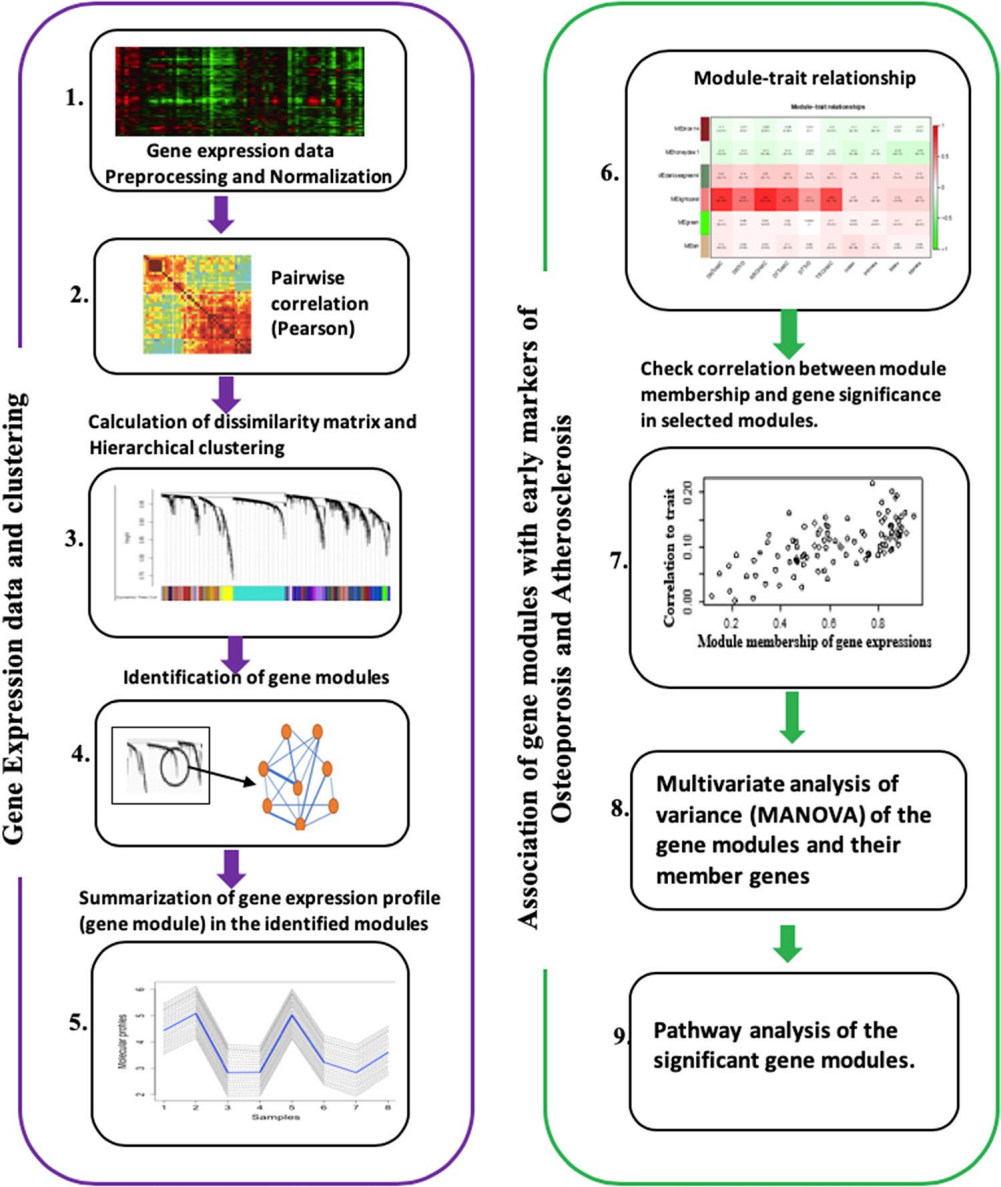
Weighted gene co-expression network analysis pipeline (Figure adapted from Fig. 1 in^7^). 1) Gene expression data analysed using Illumina HumanHT-12 version 4 Expression BeadChip. 2) Correlation matrix based on the pairwise correlations (Pearson) of the gene expression data. 3) Hierarchical clustering of the dissimilarity matrix generated from the correlation matrix. 4) Identification of gene modules based on clustering. 5) Summarization of the gene expression profile in the modules by calculating their first principal component. 6) Correlation between the modules’ summary expression profiles (representative of modules) and a set of early traits of osteoporosis and atherosclerosis. 7) Examination of the correlation between module membership and the gene significance of the gene module in the selected modules as a quality check for the modules. 8) Multivariate analysis of variance (MANOVA) of the gene modules and their member genes. 9) Pathway analysis of the member genes of the significant gene modules.

Signed weighted gene co-expression network analysis (WGCNA) implemented with R statistical software^13^ was used to identify groups of densely interconnected genes, hereafter referred to as gene modules. The analysis pipeline is illustrated in Fig. 1. The module generation method involved the calculation of Pearson’s correlation (r) for all pairwise comparisons of genes across all participants, resulting in a correlation matrix. The correlation matrix was raised to the power of 10 to generate an adjacency matrix in order to minimize noise and emphasize stronger correlations. The power was chosen using the power function implemented in the WGCNA package, to the effect that it transforms the correlation matrix to an approximately scale-free topology based on the assumption that most of the real-world biological networks are scale-free (Figure S1). The resulting adjacency matrix was used to generate a Topological Overlap Matrix (TOM) in order to incorporate network topology information in the definition of the co-expression of genes. The TOM is a similarity matrix of genes. This was transformed into a dissimilarity matrix, 1-TOM. Average linkage hierarchical clustering of the dissimilarity matrix was performed to generate a hierarchical clustering tree of genes. Next, gene modules were identified with a dynamic tree-cutting algorithm. The quality of the identified gene modules was further assessed by analysing the correlation between gene significance (GS) and module membership (MM). GS is defined as the correlation between the module's member genes and the study traits. MM is defined as the correlation between the summary expression profile of a module and its member genes. An ideal module is one where GS and MM are highly correlated, which suggests that the genes that are highly correlated with the biological marker of interest are also the important member of the analysed module. The first principal component of the expression profiles of the member genes in a module was used as a summary expression profile of the module. Pearson’s correlation coefficients (r) were calculated between the summary expression profiles of the identified gene modules and the early traits of osteoporosis and atherosclerosis. Gene modules that were significantly correlated (Bonferroni-adjusted p-value (p.adj) < 0.05) with early traits of both osteoporosis and atherosclerosis were considered as candidate modules for testing joint statistical association with the studied traits of both diseases in multivariate statistical analysis.

**Figure 2:**
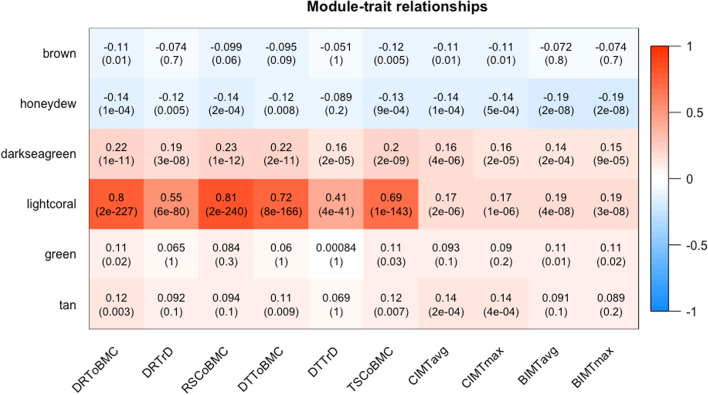
Relationships between gene co-expression modules (x-axis) and early traits (y-axis). The rows correspond to the different gene modules (named by colour) and their summary expression profile; for example, *brown* represents the summary expression profile for the module *brown*. The columns correspond to the measured early traits of osteoporosis (pQCT traits) and atherosclerosis (ultrasound traits) used in the biostatistical analyses. The values in the cells represent Pearson's correlation coefficients (r), with the associated Bonferroni-adjusted p-values in parentheses below the coefficient. The modules are named by colour, and the correlation coefficients have a colour-coding shown in the colour scale (between − 1 and + 1) on the right side of the figure. Acronyms: *DRToBMC*, distal radius total bone mineral content; *DRTrD*, distal radius trabecular bone mineral density; *RSCoBMC*, radial shaft cortical bone mineral content; *DTToBMC*, distal tibia total bone mineral content; *DTTrD*, distal tibia trabecular bone mineral density; *TSCoBMC*, tibia shaft cortical bone mineral content; *CIMTavg*, average carotid intima-media thickness; *CIMTmax*, maximum carotid intima-media thickness; *BIMTavg*, average bulbus intima-media thickness; *BIMTmax*, maximum bulbus intima-media thickness.

A multivariate analysis of variance (MANOVA) test implemented in the *car* R package was performed to test for a joint statistical association between the candidate gene modules and the studied traits of both osteoporosis and atherosclerosis. The MANOVA test was performed with three different models: **model 1** for investigating a joint association between gene modules and early traits of both osteoporosis and atherosclerosis with no covariates; **model 2,** adjusted with age, sex and body mass index (BMI); and **model 3**, which was the same as model 2 but additionally adjusted for smoking, alcohol consumption and variables related to physical activity. The early traits, one for osteoporosis and another for atherosclerosis, that had the most significant correlation with the module’s summary expression profile (Fig. 2) were chosen for the test. Biological pathway (groups of biologically related genes) analyses based on the Gene Ontology (GO)^19^, the Kyoto Encyclopedia of Genes and Genomes (KEGG)^20^ and the Disease Ontology (DO)^21^ were performed for the genes involved in the significant gene modules using *clusterProfiler* and *DOSE* R/Bioconductor packages^22,23^. In order to simplify the interpretation, significantly enriched pathways across all of the significant gene modules were clustered using the “*dendextend*” R package based on the similarity of member genes^24^.

All statistical analyses and data processing were performed using the statistical package R version 3.4.3^25^. Findings with p.adj < 0.05 were reported as significant. Considering the exploratory nature of this study, p.adj < 0.25 were reported as suggestively significant. Similar reporting approach has been used by others^26^.

## Results

### Study population characteristics

The present study is based on 1,032 participants, aged 30–45 years (56% women), from the Young Finns study 2007 follow-up. The clinical and other detailed characteristics of the study population are shown in Table 1. Number of diseases are based on self-reports^15^. The measured early traits of osteoporosis and atherosclerosis are shown in Table 2. The statistical analyses were based on four atherosclerotic and six osteoporotic traits. The gene expression levels of the study participants were profiled in 2011 follow-up.

### Identification of gene modules

The hierarchical clustering of the dissimilarity matrix defined 38 modules, containing 12–5,697 highly correlated genes. Following the standard practice in *WGCNA* analysis, the gene modules were named according to colour for downstream analysis, as shown in Fig. 2.

### Gene co-expression module trait relationships and identification of the most significant gene modules

Pearson’s correlation coefficients (r) between the modules’ summary expression profiles and the studied ten early traits were analysed. Six modules (*brown, honeydew, darkseagreen, lightcoral, green* and *tan*) were found to be significantly correlated with several of the early traits of both osteoporosis and atherosclerosis (Fig. 2). Constituent genes of the six modules are listed in Tables S1–S6. Two modules, *darkseagreen* and *lightcoral*, were significantly correlated with all of the studied early traits of both osteoporosis and atherosclerosis. Within the *lightcoral* module, the correlation values (r) between the module’s summary expression profile and different study traits varied between 0.17 and 0.81, and the Bonferroni adjusted p-values (p.adj) for these correlations were between 2 × 10^–6^ and 2 × 10^–240^. Within the *darkseagreen* module, the corresponding r was 0.14–0.23 and p.adj ≤ 2 × 10^–4^ for all traits. Similarly, the module *honeydew* was significantly but inversely correlated with all of the studied traits of atherosclerosis and with all but one (i.e. *DTTrD*) early trait of osteoporosis (r varied between -0.12 and -0.19, p.adj ≤ 0.005 for all traits). The *brown* module was significantly associated with the carotid-IMT-related variables *CIMTavg* (r = -0.11, p.adj = 0.01) and *CIMTmax* (r = -0.11, p.adj = 0.01). The same module was also significantly correlated with three of the pQCT bone measurements, the highest correlation being with *TSCoBMC* (r = -0.12, p.adj = 0.005). Similarly, the *green* and *tan* modules were significantly associated with several early traits of both osteoporosis (r ≥ 0.11, p.adj < 0.05) and atherosclerosis (r ≥ 0.11, p.adj < 0.05). All six significant gene modules (Fig. 2) had a significant correlation between GS and MM with regard to the early traits of both atherosclerosis and osteoporosis, with r ≥ 0.34 and a p-value of $\le 0.05$ (Figure S2–S7).

### Multivariate analysis of the six significant gene co-expression modules with early traits of osteoporosis and atherosclerosis

The results from the MANOVA test with the three different models (1–3) described in Sect. 2.6 are presented in Table 3. The Pillai’s Trace statistic represents the magnitude of the effect of the module’s summary expression profile on the early traits of osteoporosis and atherosclerosis. The value of the statistic ranges from 0 to 1, with higher values meaning higher effect. The F-value represents the predictive ability of a model: a higher F-value suggests higher significance of a model. All six gene modules were jointly and significantly associated with early traits of both osteoporosis and atherosclerosis in model 1 (p.adj $\le$ 3.7 × 10^−5^). Two of the six significant modules, *honeydew* and *green*, were jointly and significantly associated with early traits of both osteoporosis and atherosclerosis in models 2 and 3 (p.adj $\le$ 0.03).

**Table 3:** Multivariate analysis of variance (MANOVA) test results from association analyses between the summary expression profiles of the six significant gene modules and early traits of both osteoporosis and atherosclerosis, using three differently adjusted models (1–3), as described in the table footnote*. The most significant gene modules across all the three tested models are in bold.

Gene modules (number of genes)	Statistical models*	Pillai’s Trace	F-value	Bonferroni-adjusted p-value
*brown* (1,205)	Model 1	0.02	12.18	**5.9 × 10**^**−6**^
	Model 2	0.003	1.30	0.27
	Model 3	0.002	1.09	0.34
***honeydew (33)***	Model 1	0.05	26.17	**8.17 × 10**^**−12**^
	Model 2	0.009	4.0	**0.01**
	Model 3	0.007	4.0	**0.03**
*darkseagreen* (28)	Model 1	0.07	38.25	**2.2 × 10**^**−16**^
	Model 2	0.004	2.0	0.13
	Model 3	0.003	2.0	0.19
*lightcoral* (13)	Model 1	0.66	989.62	**2.2 × 10**^**−16**^
	Model 2	0.0004	0.23	0.79
	Model 3	0.0004	0.2	0.84
***green (3,885)***	Model 1	0.02	10.31	**3.7 × 10**^**−5**^
	Model 2	0.007	4.0	**0.03**
	Model 3	0.008	4.0	**0.02**
*Tan* (504)	Model 1	0.03	15.44	**2.5 × 10**^**−7**^
	Model 2	0.004	2.0	0.13
	Model 3	0.003	2.0	0.18

Statistical models*: MANOVA model 1 was designed to investigate the joint association between the summary expression profiles of the gene modules and early traits of both osteoporosis and atherosclerosis with no covariates. Model 2 was adjusted with age, sex and body mass index. Model 3 was the same as model 2 but additionally adjusted for smoking, alcohol consumption and variables related to physical activity.

### Multivariate analysis of the member genes of the six significant gene modules with early traits of osteoporosis and atherosclerosis

According to model 1, there were 261 genes significantly and jointly associated with early traits of both osteoporosis (*TSCoBMC*) and atherosclerosis (*CIMTavg*) in the *brown* module (Table S7). The most significant gene (p.adj = 1.2 × 10^−10^) in the *brown* module with model 1 is solute carrier family 16 member 10 (*SLC16A10*) (Table 4). Models 2 and 3 identified Zinc Finger Protein 594 (*ZNF594*) as the most significant gene in the *brown* model, with a suggestive significance level (p.adj = 0.08).

**Table 4:** Multivariate analysis of variance (MANOVA) test results from association analyses between member genes of the six significant gene modules and early traits of both osteoporosis and atherosclerosis using three differently adjusted models (1–3), as described in the table footnote*. The topmost ranking genes based on the Bonferroni-adjusted p-value for each of the three models are presented.

Gene modules	Statistical models*	Genes	Pillai’s Trace	F statistics	Bonferroni-adjusted p-value
*brown*	Model 1	SLC16A10	0.06	31	1.2 × 10^−10^
	Model 2	ZNF594	0.02	9.7	0.08
	Model 3	ZNF594	0.02	8.4	0.31
*honeydew*	Model 1	NOSIP	0.07	40	7.4 × 10^−16^
	Model 2	NOSIP	0.01	7	0.03
	Model 3	NOSIP	0.01	6	0.09
*darkseagreen*	Model 1	MPO	0.1	60	6.8 × 10^−24^
	Model 2	PTPN20	0.01	6.6	0.04
	Model 3	PTPN20	0.01	5.4	0.13
*lightcoral*	Model 1	LOC100133662	0.68	1088	1.8 × 10^−253^
	Model 2	EIF1AY	0.0007	3.52	0.39
	Model 3	EIF1AY	0.0006	2.76	0.83
*green*	Model 1	MAP7D2	0.07	41	3.3 × 10^−14^
	Model 2	GXYLT2	0.02	10.1	0.18
	Model 3	TRIM63	0.02	11.1	0.07
*tan*	Model 1	HS.412918	0.25	169	2.1 × 10^−61^
	Model 2	C17ORF28	0.02	7.7	0.23
	Model 3	ESPN	0.01	7.0	0.49

Statistical models*: MANOVA model 1 was designed to investigate joint association between the summary expression profile of the gene modules and early traits of both osteoporosis and atherosclerosis with no covariates. Model 2 was adjusted with age, sex and body mass index. Model 3 was the same as model 2 but additionally adjusted for smoking, alcohol consumption and variables related to physical activity.

According to model 1, the *honeydew* module had 31 genes significantly and jointly associated with early traits of both osteoporosis (*DRToBMC*) and atherosclerosis (*BIMTmax*) (Table S8), the most significant (p.adj = 7.4 × 10^−16^) being Nitric Oxide Synthase Interacting Protein (*NOSIP*) (Table 4). *NOSIP* was also the most significant gene of the *honeydew* module with models 2 (p.adj = 0.03) and 3 (p.adj = 0.09).

The *darkseagreen* module had 25 genes significantly and jointly associated with early traits of both osteoporosis (*RSCoBMC*) and atherosclerosis (*CIMTavg*) in model 1 (Table S9), the most significant (p.adj = 6.8 × 10^−24^) being Myeloperoxidase (*MPO*) (Table 4). Models 2 and 3 identified Protein Tyrosine Phosphatase Non-Receptor Type 20 (*PTPN20*) as the most significant gene in the *darkseagreen* module, with a p.adj of 0.04 in model 2 and p.adj of 0.13 in model 3.

Only 13 genes in the *lightcoral* module were significantly and jointly associated with early traits of both osteoporosis (*RSCoBMC*) and atherosclerosis (*BIMTmax*) in model 1 (Table S10) and none in models 2 and 3 (Table 4). The most significant gene (p.adj = 1.8 × 10^−253^) in model 1, LOC100133662, is uncharacterized.

The *green* module had 18 genes significantly and jointly associated with early traits of both osteoporosis (*DRToBMC*) and atherosclerosis (*BIMTavg*) in model 1 (Table S11), the most significant (p.adj = 3.3 × 10^−14^) being MAP7 Domain Containing 2 (*MAP7D2*) (Table 4). Model 2 identified Glucoside Xylosyltransferase 2 (*GXYLT2)* as the most significant gene, with a p.adj of 0.18, and model 3 identified Tripartite Motif Containing 63 (*TRIM63*) as the most significant gene, with a p.adj of 0.07.

The *tan* module had 132 genes significantly and jointly associated with early traits of both osteoporosis (*DRToBMC*) and atherosclerosis (*CIMTavg*) in model 1 (Table S12), the most significant (p.adj = 2.1 × 10^−61^) being an uncharacterized gene (*HS.412918*) (Table 4). Model 2 identified chromosome 17 open reading frame 28 (*C17ORF28*) as the most significant gene in the *tan* module, with a p.adj of 0.23.

### Pathway analysis of gene modules shared by early traits of osteoporosis and atherosclerosis

**Figure 3:**
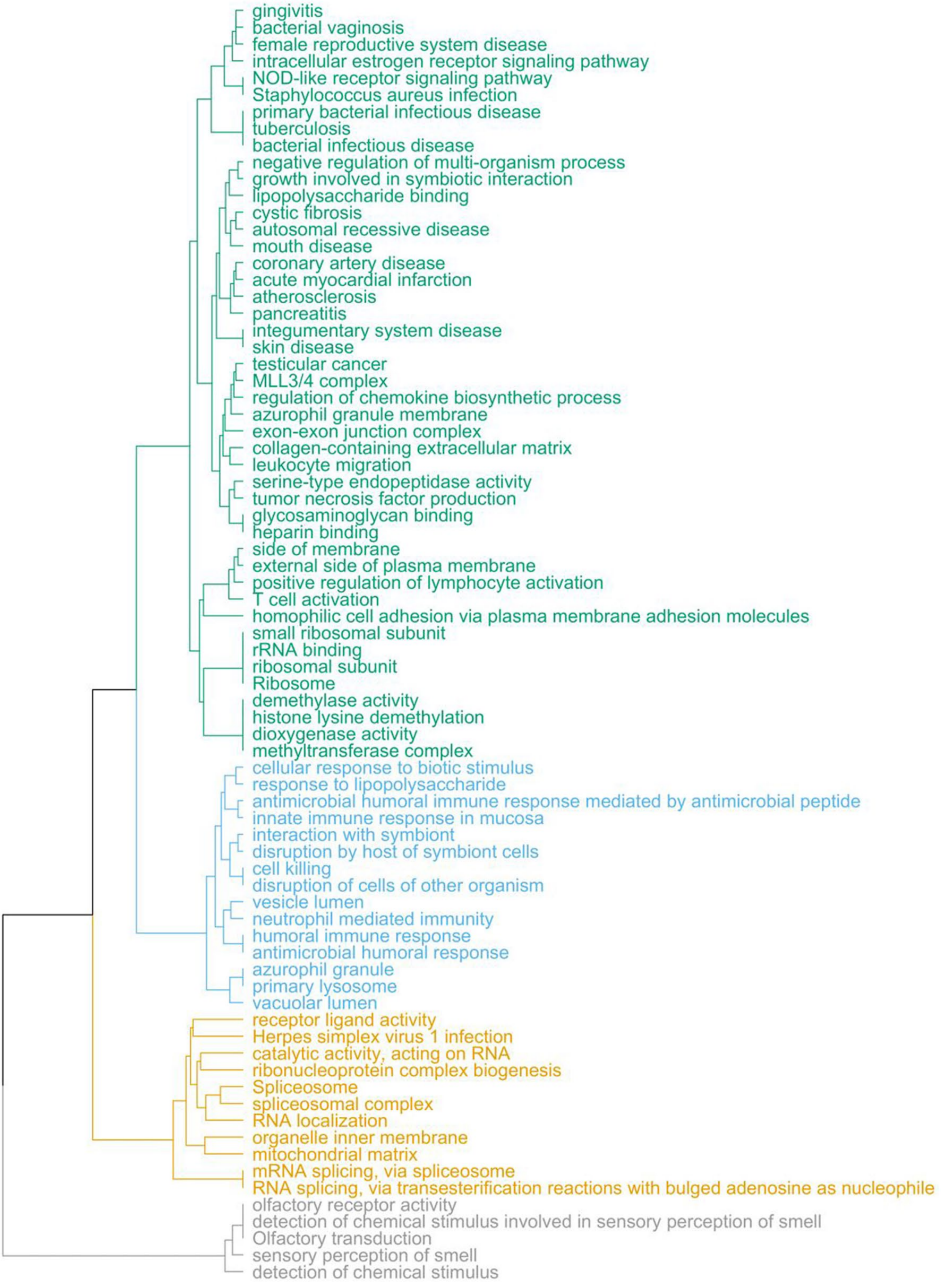
Biological pathways shared by early traits of osteoporosis and atherosclerosis. Dendrogram plot representing the hierarchical clustering of biological pathways significantly enriched (Bonferroni-adjusted p-value < 0.05) in member genes of the six significant joint gene modules (*brown4, honeydew1, darkseagreen4, lightcoral, green* and *tan*). The four clusters, from the largest to smallest in size, are represented by green, blue, orange and grey colours. Biological pathways were defined as sets of genes derived from three different knowledge bases: the Gene Ontology (GO), the Kyoto Encyclopedia of Genes and Genomes (KEGG) and the Disease Ontology (DO).

Biological pathways significantly enriched (p.adj < 0.05) across all six gene modules were clustered into four groups based on the similarity of member genes (Fig. 3). The largest cluster, coded with green in Fig. 3, mostly contained pathways related to diseases (including atherosclerosis and mouth disease) and the immune response. The second largest cluster, coded with blue in Fig. 3, contained pathways related to the immune response. The third and fourth clusters (coded with orange and grey, respectively, in Fig. 3) contained pathways respectively related to RNA metabolism and olfactory receptors.

Member genes in the *brown* module were significantly (p.adj < 0.05) enriched with nine GO terms (four biological processes, one molecular function and four cellular components) and two KEGG pathways (Table S13). Eight of the nine pathways belonged to the orange cluster, representing RNA-metabolism-related pathways (Fig. 3). Genes in the *honeydew* module were significantly enriched with 4 GO pathways (2 biological processes and 2 cellular components) (Table S14), all of which were related to the immune response represented by the green cluster (Fig. 3). Genes from the *darkseagreen* module were significantly enriched with 27 GO pathways (17 biological processes, 4 molecular functions and 6 cellular components), 2 KEGG pathways and 15 DO pathways (Table S15). Most of these pathways were related to the immune response and diseases (represented by the green cluster in Fig. 3). The *lightcoral* module contains genes that were significantly enriched with 8 GO pathways (1 biological processes, 3 molecular functions and 4 cellular components), 1 KEGG pathway and 1 DO pathway (Table S16). All the pathways in the *lightcoral* module belonged to the green cluster (Fig. 3). The *green* module was enriched with 6 GO pathways (4 biological processes and 2 molecular functions) and 1 KEGG pathway (Table S17). The pathways are related to olfactory receptors and were represented in the grey cluster (Fig. 3).

## Discussion

We performed a system-level analysis of genome-wide gene expression data to identify whole blood transcriptomic modules and the enriched biological pathways shared by early traits of osteoporosis (pQCT bone measurements) and atherosclerosis (ultrasound carotid IMT). Genome-wide expression levels from whole blood represent the average gene expression levels across all cells in whole blood and, therefore, facilitate the examination of the general expression pattern associated with a phenotype being studied. The whole blood gene expression profile recapitulates the biological processes in bone marrow, as immune cells within blood migrate back and forth between blood and bone marrow^27^ and are known to influence bone homeostasis^28^. Furthermore, a whole-blood-based approach can provide biomarkers that are easily assessable and non-invasive as opposed to bone and vascular tissue.

We identified six gene modules jointly and significantly associated with the studied traits of both diseases. Two of the six gene modules, *green* and *honeydew*, were jointly and significantly associated with the studied traits of both diseases after adjustment with age, sex, body mass index, smoking habit, alcohol consumption and physical exercise covariates. Another two modules, *darkseagreen* and *tan,* were jointly associated with the studied traits of both diseases after otherwise similar statistical adjustments, but with suggestive significance. A biological pathway analysis of the six gene modules identified several statistically significant biological pathways jointly associated with the studied traits of both diseases. Detailed gene-level analysis of the modules identified the three most significant genes (*NOSIP* from *honeydew1* module, *GXYLT2* and *TRIM63* from *green* module) jointly associated with the early traits of the diseases. The three genes have been identified by previous studies as being independently associated with osteoporosis^29–31^ and/or atherosclerosis^32,33^, thus validating our findings. However, to the best of our knowledge, this is the first study to report their joint association with early traits of both the diseases.

The biological processes (detection of chemical stimulus, sensory perception of smell and detection of chemical stimulus involved in the sensory perception of smell), molecular function (olfactory receptor activity), and the KEGG (olfactory transduction) pathway enriched in the *green* module mostly contained olfactory receptor genes that belong to the G-protein-coupled receptor gene family. Even though olfactory receptor genes are traditionally known to be responsible for detecting odours and initiating the signalling cascade, various studies have shown their diverse physiological functions in other tissues, such as the testes^34^, lungs^35^, the brain^36^ and the heart^37^. Studies have indicated that olfactory receptors play a critical role in lipid metabolism^38^ and regulate heart function^39^. Bone morphogenetic proteins act as a regulator during bone formation and repair^40^. Additionally, bone morphogenetic proteins are suggested to promote the survival of olfactory receptors’ neurons^41^. Therefore, we speculate that olfactory receptors have an important role in bone remodelling. The green module was also enriched with a receptor ligand activity (molecular function) pathway that contains genes with anti-inflammatory and anti-atherogenic properties, such as Adiponectin (*ADIPOQ*)^42^. Adiponectin prevents atherosclerosis by decreasing oxidative stress, total cholesterol, triglycerides and low-density lipoprotein-cholesterol^43^. Adiponectin also increases bone mass through the activation of osteoblasts and suppression of osteoclasts^44^. Homophilic cell adhesion via plasma membrane adhesion molecules, a biological process enriched in the green module*,* contained genes such as the polio virus receptor (*PVR*). *PVR* plays an important role in inflammatory process during atherosclerosis via leukocytes movement across the endothelium^45^. Furthermore, studies indicate that PVR-mediated signalling inhibits osteoclast formation^46^.

The biological process, T-cell activation, enriched in the *honeydew* module plays an important role in the development of both osteoporosis^47^ and atherosclerosis^48^. Similarly, the other enriched biological process, positive regulation of lymphocyte activation, is in line with existing literature, as B-lymphocytes are known to regulate the RANK-RANKL-OPG pathway that plays a role in basal bone homeostasis, osteoclast formation and the regulation of bone resorption^49^. Both biological processes are also important for atherosclerosis because the disease involves immune cells such as macrophages and T-lymphocytes^50^.

The module *darkseagreen* was enriched with 15 biological pathways based on the Disease Ontology (DO), a knowledge base of human diseases. Three of the DO pathways (coronary artery disease, acute myocardial infarction and atherosclerosis) are related to atherosclerosis, and one of the DO pathways, *mouth disease,* is related to bone disease^51^. There were 17 biological pathways related to biological processes, most of which were related to the immune system, which is central to both bone and vascular health.

The most significant genes jointly associated with early traits of both osteoporosis and atherosclerosis were *NOSIP* (module: *honeydew1*), *GXYLT2* (module: *green)* and *TRIM63* (module: *green*). NOSIP, expressed mostly in musculoskeletal muscle and also in bone-derived and whole blood cells, is related to the metabolism of nitric oxide, which plays a crucial role in the pathogenesis of both osteoporosis^29^ and atherosclerosis^32^. A moderate induction of nitric oxide promotes bone resorption, while constitutive production of nitric oxide induces the proliferation of osteoblast-like cells. *GXYLT2* is expressed in whole blood (immune cells) and several other tissues as well. The gene encodes for xylosyltransferase, which plays a role in the biosynthesis of glycosaminoglycan chains, an important constituent of proteoglycans. Proteoglycans are among several extracellular matrix molecules that congregate in atherosclerosis lesions^33^ and regulate the osteolytic process^30^. The third significant gene, *TRIM63* (Tripartite Motif Containing 63), also known as *MURF1* (muscle-specific ring finger protein 1), is mostly expressed in skeletal muscle but moderately also in other tissues, such as whole blood (immune cells) and osteoblastic cells, when induced by glucocorticoids. The expression of *TRIM63* has been shown to promote osteoblastic cell differentiation and suppress the proliferation of osteoblastic cells^31^. While *TRIM63* is known to play a role in the regulation of cardiac hypertrophy^52^, its role in atherosclerosis is unclear. All three genes are related to the immune system, which plays an important role in the development of both osteoporosis and atherosclerosis. TRIM63 is related to the innate immune system. NOSIP is involved in the metabolism of nitric oxide, which is a key player in the immune system. GXYLT2 plays a role in transferase activity, which also affects immune function.

There were certain limitations to the study. The study was based on a relatively young population cohort with an early phase of cardiovascular disease and osteoporosis and very few clinically diagnosed cases. Therefore, the early traits of the diseases used in the study were positively correlated, as shown in our previous study^7^. A positive correlation between the early traits of osteoporosis and atherosclerosis, such as increasing bone density with an increase in CIMT, is counterintuitive for the comorbidity hypothesis. However, we and others^53^ suggest that the positive correlation might be reflecting the shared biological mechanisms between bone and vascular tissue during normal growth and development. The study was thus focused on identifying transcriptomic biomarkers from whole blood that are associated with early traits of bone and vascular health. We speculate that the direction of the association between these two sets of traits representing osteoporotic and atherosclerotic comorbidity and, consequently, also the association with the identified transcriptomic biomarkers, may change systematically due to unhealthy lifestyle choices, similarly to the phenomenon known as “decoherence”^54^. The joint association between the significant modules (*green and honeydew*) and the early traits of the diseases is weak, albeit significant, perhaps due to the relatively young and healthy cohort, as described above. We, however, believe that the results show a suggestive joint association between the modules and traits of the diseases that warrants further research in a case–control setting that includes participants with clinically diagnosed osteoporosis and atherosclerosis. Another limitation of the study was the time difference (four years) between the measurement of the early traits of the diseases and the transcriptomic profile of the study participants. The study was based on the assumption that there is no substantial change in bone and carotid artery measurements over a four-year period among a healthy young population. Also, all study participants are of European origin. Further research in a case–control setting with populations of different ethnicities are needed. This study was based on microarray technology as RNA-Seq technology was still too expensive at the time of follow-up (year 2011) for a large epidemiological study such as the one in this study.

## Summary and conclusion

There is a lack of omics-based studies investigating osteoporosis and atherosclerosis comorbidity in the literature despite strong and clear indications from several studies that the diseases are comorbid. We performed system-level analysis of joint associations between early traits of both diseases and transcriptomics modules. The study identified six genome-wide gene co-expression modules, and several enriched biological pathways within the modules, that are significantly and jointly associated with early traits of both the diseases, supporting our comorbidity hypothesis. Detailed analysis of the gene co-expression modules identified three genes (*NOSIP, GXYLT2 and TRIM63*) that might play an important role in developing dual-purpose prevention methods.

## Supplementary Information

Supplementary Information

Supplementary Information

## Data Availability

The dataset supporting the conclusions of this article were obtained from the Cardiovascular Risk in Young Finns study which comprises health related participant data. The use of data is restricted under the regulations on professional secrecy (Act on the Openness of Government Activities, 612/1999) and on sensitive personal data (Personal Data Act, 523/1999, implementing the EU data protection directive 95/46/EC). Due to these restrictions, the data cannot be stored in public repositories or otherwise made publicly available. Data access may be permitted on a case-by-case basis upon request only. Data sharing outside the group is done in collaboration with YFS group and requires a data-sharing agreement. Investigators can submit an expression of interest to the chairman of the publication committee, Prof Mika Kähönen (Tampere University, Finland) and Prof Terho Lehtimäki (Tampere University, Finland).
