# Supplementary material for: Modular genome-wide gene expression architecture shared by early traits of osteoporosis and atherosclerosis in the Young Finns Study
Source: Sci Rep. 2021 Mar 29;11:7111. doi: 10.1038/s41598-021-86536-0 (PMC8007808; doi:10.1038/s41598-021-86536-0)
Supplement: Supplementary file 1 — Supplementary Information [file 41598_2021_86536_MOESM1_ESM.docx]

**Modular genome-wide gene expression architecture shared by early traits of osteoporosis and atherosclerosis in the Young Finns Study**

Binisha H. Mishra^1,2,3^, Pashupati P. Mishra^1,2,3^*,* Emma Raitoharju^1,2,3^, Saara Marttila^1,2,3,4^, Nina Mononen^1,2,3^, Harri Sievänen^5^, Jorma Viikari^6,7^, Markus Juonala^6,7,8^, Marika Laaksonen^9^, Nina Hutri-Kähönen^10^, Mika Kähönen^2,11^, Olli T. Raitakari^8,12,13^, Terho Lehtimäki^1,2,3^

^1^Department of Clinical Chemistry, Faculty of Medicine and Health Technology, Tampere University, Tampere, Finland

^2^Finnish Cardiovascular Research Center Tampere, Faculty of Medicine and Health Technology, Tampere University, Tampere, Finland

^3^Department of Clinical Chemistry, Fimlab Laboratories, Tampere, Finland

^4^Gerontology Research Center (GEREC), Tampere University, Tampere, Finland

^5^The UKK Institute for Health Promotion Research, Tampere, Finland

^6^Department of Medicine, University of Turku, Turku, Finland

^7^Division of Medicine, Turku University Hospital, Turku, Finland

^8^Research Centre of Applied and Preventive Cardiovascular Medicine, University of Turku, Turku, Finland

^9^Fazer Lab Research, Oy Karl Fazer Ab, Helsinki, Finland

^10^Department of Paediatrics, Tampere University Hospital, Faculty of Medicine and Health Technology, Tampere University, Tampere, Finland

^11^Department of Clinical Physiology, Tampere University Hospital, Tampere, Finland.

^12^Department of Clinical Physiology and Nuclear Medicine, Turku University Hospital, Turku, Finland

^13^Centre for Population Health Research, University of Turku and Turku University Hospital, Turku, Finland

**Correspondence to:**

Binisha H. Mishra, M.Sc., PhD student (binisha.hamalmishra@tuni.fi)

Faculty of Medicine and Health Technology,

Department of Clinical Chemistry, Tampere University,

Fimlab Laboratories,

and Finnish Cardiovascular Research Center Tampere.

**Supplementary figures**

**Figure S1.** Determination of soft-thresholding power in weighted gene co-expression network analysis. Summary network indices (scale free topology and mean connectivity) (y-axis) as functions of the soft thresholding power (x-axis). The plot indicates that approximate scale-free topology is attained around the soft-thresholding power of 10.

**Figure S2.** Scatter plots of gene significance (GS) vs module membership (MM) in the brown4 module. The left panel corresponds to subclinical markers of osteoporosis and the right panel to subclinical markers of atherosclerosis. Abbreviations: TSCoMC, Total mineral content in the tibia shaft's cortical bone: imtav, carotid intima-media thickness (average).

**Figure S3**. Scatter plots of gene significance (GS) vs module membership (MM) in the honeydew1 module. The left panel corresponds to subclinical markers of osteoporosis and the right panel to subclinical markers of atherosclerosis. Abbreviations: DRToMC, Total mineral content of the distal radius: bbav, bulbus intima-media thickness (average).

**Figure S4.** Scatter plots of gene significance (GS) vs module membership (MM) in the darkseagreen4 module. The left panel corresponds to subclinical markers of osteoporosis and the right panel to subclinical markers of atherosclerosis. Abbreviations: RSCoMC, Total mineral content in the radial shaft's cortical bone: imtav, carotid intima-media thickness (average).

**Figure S5**. Scatter plots of gene significance (GS) vs module membership (MM) in the lightcoral module. The left panel corresponds to subclinical markers of osteoporosis and the right panel to subclinical markers of atherosclerosis. Abbreviations: RSCoMC, Total mineral content in the radial shaft's cortical bone: bbmax, bulbus intima-media thickness (maximum).

**Figure S6.** Scatter plots of gene significance (GS) vs module membership (MM) in the green module. The left panel corresponds to subclinical markers of osteoporosis and the right panel to subclinical markers of atherosclerosis. Abbreviations: DRToMC, Total mineral content of the distal radius: bbmax, bulbus intima-media thickness (maximum).

**Figure S7**. Scatter plots of gene significance (GS) vs module membership (MM) in the green module. The left panel corresponds to subclinical markers of osteoporosis and the right panel to subclinical markers of atherosclerosis. Abbreviations: DRToMC, Total mineral content of the distal radius: imtav, carotid intima-media thickness (average).
